# Ipsilesional Mu Rhythm Desynchronization Correlates With Improvements in Affected Hand Grip Strength and Functional Connectivity in Sensorimotor Cortices Following BCI-FES Intervention for Upper Extremity in Stroke Survivors

**DOI:** 10.3389/fnhum.2021.725645

**Published:** 2021-10-28

**Authors:** Alexander B. Remsik, Klevest Gjini, Leroy Williams, Peter L. E. van Kan, Shawna Gloe, Erik Bjorklund, Cameron A. Rivera, Sophia Romero, Brittany M. Young, Veena A. Nair, Kristin E. Caldera, Justin C. Williams, Vivek Prabhakaran

**Affiliations:** ^1^Department of Radiology, University of Wisconsin–Madison, Madison, WI, United States; ^2^Department of Kinesiology, University of Wisconsin–Madison, Madison, WI, United States; ^3^Institute for Clinical and Translational Research, University of Wisconsin–Madison, Madison, WI, United States; ^4^Department of Neurology, University of Wisconsin–Madison, Madison, WI, United States; ^5^Department of Educational Psychology, University of Wisconsin–Madison, Madison, WI, United States; ^6^Center for Women’s Health Research, University of Wisconsin–Madison, Madison, WI, United States; ^7^Neuroscience Training Program, University of Wisconsin School of Medicine and Public Health, Madison, WI, United States; ^8^Clinical Neuroengineering Training Program, University of Wisconsin–Madison, Madison, WI, United States; ^9^Medical Scientist Training Program, University of Wisconsin School of Medicine and Public Health, Madison, WI, United States; ^10^Department of Biomedical Engineering, University of Wisconsin–Madison, Madison, WI, United States; ^11^Department of Neurological Surgery, University of Wisconsin–Madison, Madison, WI, United States; ^12^Department of Psychiatry, University of Wisconsin–Madison, Madison, WI, United States; ^13^Department of Psychology, University of Wisconsin–Madison, Madison, WI, United States

**Keywords:** brain-computer interface, sensorimotor rhythm, Mu, Beta, stroke, motor function, iCoh

## Abstract

Stroke is a leading cause of acquired long-term upper extremity motor disability. Current standard of care trajectories fail to deliver sufficient motor rehabilitation to stroke survivors. Recent research suggests that use of brain-computer interface (BCI) devices improves motor function in stroke survivors, regardless of stroke severity and chronicity, and may induce and/or facilitate neuroplastic changes associated with motor rehabilitation. The present sub analyses of ongoing crossover-controlled trial NCT02098265 examine first whether, during movements of the affected hand compared to rest, ipsilesional Mu rhythm desynchronization of cerebral cortical sensorimotor areas [Brodmann’s areas (BA) 1-7] is localized and tracks with changes in grip force strength. Secondly, we test the hypothesis that BCI intervention results in changes in frequency-specific directional flow of information transmission (direct path functional connectivity) in BA 1-7 by measuring changes in isolated effective coherence (iCoh) between cerebral cortical sensorimotor areas thought to relate to electrophysiological signatures of motor actions and motor learning. A sample of 16 stroke survivors with right hemisphere lesions (left hand motor impairment), received a maximum of 18–30 h of BCI intervention. Electroencephalograms were recorded during intervention sessions while outcome measures of motor function and capacity were assessed at baseline and completion of intervention. Greater desynchronization of Mu rhythm, during movements of the impaired hand compared to rest, were primarily localized to ipsilesional sensorimotor cortices (BA 1-7). In addition, increased Mu desynchronization in the ipsilesional primary motor cortex, Post vs. Pre BCI intervention, correlated significantly with improvements in hand function as assessed by grip force measurements. Moreover, the results show a significant change in the direction of causal information flow, as measured by iCoh, toward the ipsilesional motor (BA 4) and ipsilesional premotor cortices (BA 6) during BCI intervention. Significant iCoh increases from ipsilesional BA 4 to ipsilesional BA 6 were observed in both Mu [8–12 Hz] and Beta [18–26 Hz] frequency ranges. In summary, the present results are indicative of improvements in motor capacity and behavior, and they are consistent with the view that BCI-FES intervention improves functional motor capacity of the ipsilesional hemisphere and the impaired hand.

## Introduction

Stroke is a leading cause of acquired upper extremity (UE) motor disability and many survivors are left with persistent upper extremity motor impairments requiring rehabilitation (Benjamin et al., 2017, 2019). In the United States alone, on average, every 40 seconds someone suffers a stroke (Benjamin et al., 2019). About 6 months after stroke insult, approximately half of stroke survivors continue to suffer residual motor deficit (Benjamin et al., 2017). Stroke burden on the United States economy, by 2050, is expected to be above $2.2 trillion (Benjamin et al., 2017). Despite advances in acute stroke care, the estimated direct and indirect costs of stroke continue to escalate and are disproportionately associated with long-term care and rehabilitation (Benjamin et al., 2017, 2019).

Electroencephalogram-based brain-computer interface (BCI) intervention has been proposed as a novel intervention tool (Pfurtscheller and Berghold, 1989; Pfurtscheller et al., 1997, 2005; Neuper and Pfurtscheller, 2001; Wolpaw et al., 2002; Leuthardt et al., 2004; Schalk et al., 2004; Birbaumer, 2006; Birbaumer et al., 2006; Wilson et al., 2009; Prasad et al., 2010; Schalk and Mellinger, 2010; Bundy et al., 2012; Babaiasl et al., 2016; Irimia et al., 2016; Young et al., 2016; Mazrooyisebdani et al., 2018; Mohanty et al., 2018a, b; Remsik et al., 2018; Remsik A. B. et al., 2019) capable of enhancing motor recovery post-stroke. The neural mechanisms underlying BCI’s effect on motor rehabilitation, either through neural plasticity or otherwise, are not well understood. BCIs are a promising supplement to existing means of neurorehabilitation but may also function as tools that provide insight into the sensorimotor processes underlying motor function and motor learning in either healthy or stroke-lesioned brains. Damage to the input (afferent) or output (efferent) pathways of the sensorimotor system creates a demand for reorganization of existing neural network functions (with respect to completion of behavioral goals) (Nudo, 2011; Nudo and McNeal, 2013). BCIs may induce or facilitate neuroplasticity by strengthening such connections between brain areas (Song et al., 2014, 2015; Young et al., 2014c; Biasiucci et al., 2018; Remsik A. B. et al., 2019).

EEG activity recorded from sensorimotor cortices (BA 1-7) of each hemisphere desynchronizes with imagined and attempted movements, and preparation of movement. This phenomenon is known as event-related desynchronization (ERD) (Pfurtscheller and Berghold, 1989; Pfurtscheller et al., 1997, 2005; Pfurtscheller, 1999; Pfurtscheller and Lopes da Silva, 1999; Neuper and Pfurtscheller, 2001; Pineda, 2005; Neuper et al., 2006; Nam et al., 2011). Specific frequency bands are associated with components of event-related motor behaviors (Pfurtscheller et al., 1997; Neuper and Pfurtscheller, 2001; Pineda, 2005; Nam et al., 2011; Remsik A. B. et al., 2019). When healthy individuals plan and execute purposeful movements, Mu rhythms of the contralateral sensorimotor cortices are desynchronized and attenuated (Pfurtscheller et al., 1997) and increased presence of Beta rhythm ERD is associated with motor command output and control (Pfurtscheller and Lopes da Silva, 1999; Pineda, 2005; Nam et al., 2011). ERD and event related synchronicity (ERS) were key components in the use and development of early EEG-based BCI motor rehabilitations (Wolpaw et al., 1991, 2002; Pfurtscheller et al., 1997, 2005; Pfurtscheller, 1999; McFarland et al., 2000; Neuper and Pfurtscheller, 2001; Leuthardt et al., 2004; Schalk et al., 2004; Neuper et al., 2006). Thus, ERD/ERS provide measures of volitional movement-related brain activity that can be decoded by a BCI to control a device, such as a prosthetic limb, or an output command, such as a cursor on a computer screen (Pfurtscheller et al., 1997; Leuthardt et al., 2004; Neuper et al., 2006; Wilson et al., 2009).

In human subjects, at frequencies around 10–20 Hz, Mu and Beta sensorimotor rhythms (SMRs) are recorded exclusively over cortical sensorimotor areas (BA 1-7) (Pfurtscheller et al., 1997; Birbaumer, 2006; Birbaumer et al., 2006). Two basic strategies, motor imagery and attempted movement (Wolpaw et al., 1991, 2002; Wilson et al., 2009; Nam et al., 2011; Ortner et al., 2012; Wilson et al., 2012; Song et al., 2014, 2015; Young et al., 2014a,b,c,d, 2015, 2016; Nair et al., 2015; Irimia et al., 2016; Remsik et al., 2016, 2018; Mazrooyisebdani et al., 2018; Mohanty et al., 2018a; Remsik A. B. et al., 2019) as well as various therapeutic adjuvant approaches (e.g., BCI-FES) (Biasiucci et al., 2018) have been introduced for motor rehabilitation in stroke patients. Both approaches record SMRs as input signals (electrophysiological recordings by the EEG cap) to the BCI from overlapping neural architecture. This protocol was designed to utilize attempted hand movements during the intervention following the logic that a motor therapy designed to rehabilitate volitional motor function of the affected UE should utilize voluntary attempted movements of an impaired hand in a continuous effort to improve the participant’s UE capacity and performance.

The present sub analyses of ongoing crossover-controlled trial NCT02098265 examine first whether, during movements of the affected hand compared to rest, ipsilesional Mu rhythm desynchronization (Pfurtscheller and Berghold, 1989; Pfurtscheller et al., 1997; Neuper and Pfurtscheller, 2001; Pineda, 2005; Nam et al., 2011) of cerebral cortical sensorimotor areas (Brodmann’s areas (BA) 1-7) (Brodmann, 1909) is localized and tracks with changes in grip force strength. Secondly, we test the hypothesis that BCI intervention results in changes in frequency-specific directional flow of information transmission (direct path functional connectivity) in BA 1-7 by measuring changes in isolated effective coherence, iCoh (Pascual-Marqui et al., 2014; Kitaura et al., 2017), between cerebral cortical sensorimotor areas thought to relate to electrophysiological signatures of motor actions and motor learning. Brief reports of some of the results have been published (Remsik A. et al., 2019; Remsik A. et al., 2020; Remsik A. B. et al., 2020).

## Materials and Methods

### Participant Population

Participants were recruited from the greater Madison, Wisconsin, United States area as part of an on-going cross-over controlled prospective randomized rehabilitation study investigating interventional BCI in upper extremity (UE) motor function impairment resulting from stroke. This study is approved by the University of Wisconsin Health Sciences Institutional Review Board (Study ID 20150469). All subjects provided informed written consent upon enrollment.

### Study Design and Procedures

The BCI system and intervention procedures were consistent with previously published works, Wilson et al. (2009, 2012) and Song et al. (2014) using BCI2000 software (Schalk et al., 2004) version 2 with in-house modifications for input from a 16-channel EEG cap and amplifier (Guger Technologies) and integration with a ball and target gaming visual display (Wilson et al., 2009) and functional electrical stimulation (FES) adjuvant (Popovic et al., 2009; Takahashi et al., 2012; Biasiucci et al., 2018). A more detailed and complete description of the session and run procedures used in data acquisition and analysis reported in this manuscript, as well as a description of the functional electrical stimulation protocol and justification for how this approach further improves the effects of BCI have been published previously (Remsik A. B. et al., 2019).

Sixteen right hemisphere stroke survivors (8 female, age = 62.5 ± 12.7 years [mean ± SD]) (Table 1) participated in 9–15 sessions (13.9 ± 1.28 [mean ± SD]; following removal of artifact EEG data: 11.1 ± 3.87 [mean ± SD]) with the BCI. Participants executed hand movements in response to visual cues displayed on a computer screen concordantly with corresponding audio instructions (e.g., Left, Right, Rest). One session (or run) of the cursor and target task consisted of 10 trials, or attempts, during which the participant attempted to drive a virtual cursor across the display screen into the target space using voluntary modulation of their sensorimotor rhythms (i.e., satisfaction of BCI classifier) before the trial timed out. BCI performance was measured as the number of successful trials (i.e., hits compared to misses) “out of a possible 10” attempts in any one run. This metric in included in Table 1 as “Average BCI Performance.”

**TABLE 1:** Demographics of *n* = 16 stroke survivors.

Participant	Lesion location	Time since stroke (days)	Age range	Sex	Total BCI runs	Average BCI performance	ARAT baseline	ARAT change	Hand grip strength (lb) baseline	Hand grip change (lb)
1	L. Lateral Medulla	160	45–49	M	502	6.094	3	−1	0	0
2	R. MCA	490	50–54	M	651	7.043	3	1	23.33	−10
3	Leg/periventricular white, MHR	658	75–79	M	488	5.76	57	0	51	−3
4	R-PLIC putamen	2,723	65–69	M	381	5.83	23	17	8.33	15.33
5	R. prefrontal, midfrontal, temporal	197	70–74	F	318	5.23	0	0	0	0
6	R. White matter	94	60–64	F	514	4.65	56	1	22.33	10
7	R. frontal parietal	2,645	40–44	F	464	4.9	7	0	1.33	5
8	R. Frontal-temporal-occipital	588	55–59	F	471	5.47	3	1	17	8.67
9	R. Anterior Temporal lobe; fronto-parietal regions	452	45–49	M	372	5.49	0	2	0	0
10	R. MCA/R. Frontoparietal infarct	3,017	45–49	F	392	6.92	3	1	0	0
11	R. MCA/R. Temporalfrontal-parietal	790	70–74	F	360	7.92	3	−3	0	0
12	R occipital	631	80–84	M	451	5.84	57	0	6.33	8
13	R. MCA/ACA	5,115	75–79	M	570	5.52	9	2	0	0
14	R frontal lobe R frontal hemorrhagic infarct	392	60–64	F	381	5.83	3	2	0	0.33
15	R VAOA, subarachnoid hemorrhage	2,767	55–59	F	334	5.83	57	0	49	−7.33
16	R. MCA	783	70–74	M	355	5.18	0	0	0	0
Mean		1343			437.75	5.85	17.75	1.44	11.17	1.69
SD		1449.64			92.47	0.84	23.88	4.34	17.22	6.41

*One session (or run) of the cursor and target task consisted of 10 trials, or attempts, during which the participant attempted to drive a virtual cursor across the display screen into the target space using voluntary modulation of their sensorimotor rhythms (i.e., satisfaction of BCI classifier). BCI performance was measured as the number of successful trials (i.e., hits compared to misses) “out of a possible 10” attempts in any one run. Action Research Arm Test (ARAT) scores affected (i.e., left) hand motor impairment on a scale of 0–57; 57 indicating no measurable upper extremity motor impairment. Hand Grip Strength measured by dynamometer in pounds (lb.) of maximal whole hand grasp.*

The BCI classifier was defined in “screening” sessions (Pre and Post BCI intervention). Screening sessions contained two runs, each consisting of 15 trials for rest, left-hand, and right-hand movements (i.e., 5 trials for each of the three conditions, the order of trials in a run was random). Only the rest and left-hand movement trials are considered here.

From the larger NCT02098265 cohort, only participants with left hand motor impairment were selected for these sub analyses in an effort to control for homogeneity of stroke-related upper extremity impairment, and language or communicative deficits that might interfere with comprehension of the BCI task requirements or task execution.

#### Preprocessing of the Scalp EEG Data

Various EEG-signal processing techniques were used to estimate and further evaluate the spectral perturbations recorded over the course of BCI intervention. EEG data analysis and statistics were consistent with methods detailed previously (Remsik A. B. et al., 2019).

**FIGURE 1:**
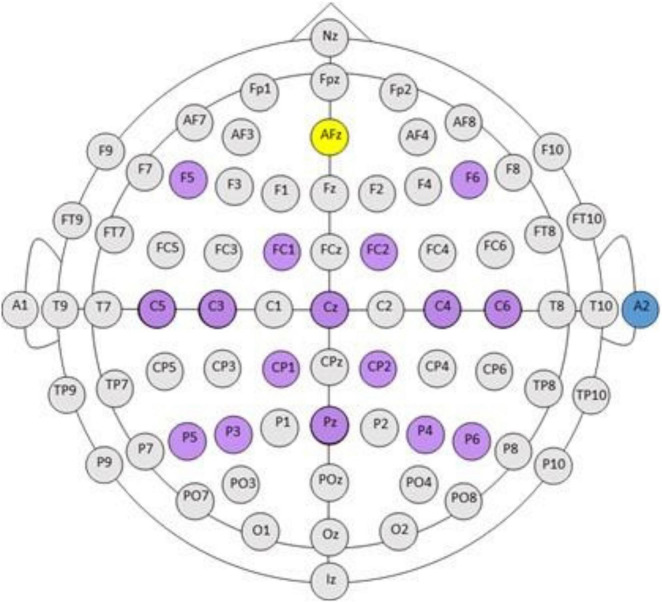
BCI cap array. International 10–20 system for standardized EEG electrode locations on the head: C, central; P, parietal; T, temporal; F, frontal; Fp, pre-frontal; O, occipital. Electrode array and cap arrangement for all *n* = 16 participants is indicated in purple against the standard 10–20 electrode array head map. AFz (yellow) is the ground electrode. A2 (blue) electrode is the reference electrode, placed on the back of the right ear. Please note, electrode arrangement is designed for increased density around cortical sensorimotor areas.

EEG electrodes were positioned according to the standard 10-20 system, grounded to Fpz, and referenced to an ear electrode placed on the back of the participant’s right ear. Electrodes in the cap arrangement are highlight in purple in Figure 1 and contain electrodes F5, F6, FC1, FC2, C5, C3, Cz, C4, C6, CP1, CP2, P5, P4, Pz, P4, P6, A2. Inputs to the BCI electrodes over the sensorimotor cortices including, C3, C4, and Cz, were recorded in every session (Figure 1). EEG preprocessing steps include: (1) Digital filtering with a high-pass filter cutoff of 4 Hz, and a low-pass filter cutoff of 30 Hz. (2) Extraction and grouping of trials according to condition (rest, left/impaired hand movement, movement type (attempted), and the screening phase (Pre, Post intervention). This resulted in 10 trials for each of condition/movement/screening phase combinations. (3) Identification and repair of bad (noisy) channels (via spline interpolation), followed by the removal of the three trials with the highest variance. The channel was identified as bad (noisy, poor connection, etc.) if the variance was <10 or >250 μV^2^ in more than three trials. The units of the variance were those of the data squared: as the EEG data units were in μV, the variance units were squared μV. If more than four channels were identified as bad, the data for that session were removed from further analysis. At session level, this step resulted in 28 s of EEG data (7 trials × 4 s) for each condition/movement/screening phase combination set. (4) An average-reference montage was applied to the data (i.e., re-referencing from the original monopolar recordings). (5) Analyses with Fourier transforms computed using a multi-taper method (Thomson, 1982; Mitra and Pesaran, 1999) at a 0.25 Hz resolution. This resulted in estimates of absolute spectral power sampled for every 1 Hz bin during the interval of 4–30 Hz, and cross-spectral density. The trial length was 4 s and the resolution of Fourier transforms was 1/4 = 0.25 Hz.

#### Use of Brodmann Areas

**FIGURE 2:**
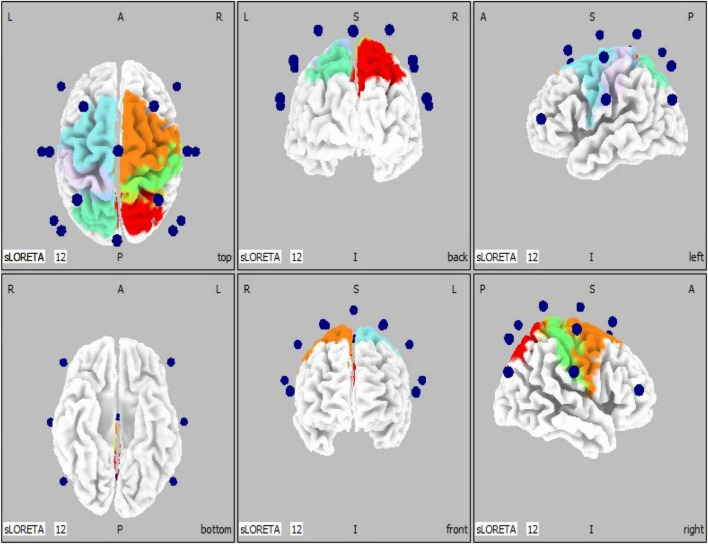
EEG electrode locations (*n* = 16, blue spheres) are shown superimposed on a generic head model (left, middle, and right panels show top/bottom, back/front, and left/right views, respectively). BA, Brodmann’s areas. Regions of interests (*n* = 10) are defined as follows: ROI #1 = left BA 1-3 (primary somatosensory cortices, light purple); ROI #2 = left BA 4 (primary motor cortex, light blue); ROI #3 = left BA 5 (somatosensory association area, cyan); ROI #4 = left BA 6 (premotor cortices, light blue); ROI #5 = left BA 7 (visuomotor coordination area, cyan); ROI #6 = right BA 1-3 (primary somatosensory cortices, green); ROI #7 = right BA 4 (primary motor cortex, orange); ROI #8 = right BA 5 (somatosensory association area, red); ROI #9 = right BA 6 (premotor cortices, orange); ROI #10 = right BA 7 (visuomotor coordination area, red).

To better illustrate the cerebral cortical sensorimotor areas represented by the co-registration of the 16-channel spectral EEG recordings to a three-dimensional head model space, we implemented ten regions of interest (ROIs). These ROIs are based on Brodmann’s areas 1–7 (Brodmann, 1909), and coincide with the electrode placement in the standardized EEG cap (Figure 2). Regions of interests were defined as follows: ROI #1 = left BA 1-3 (primary somatosensory cortices); ROI #2 = left BA 4 (primary motor cortex); ROI #3 = left BA 5 (somatosensory association area); ROI #4 = left BA 6 (premotor cortices); ROI #5 = left BA 7 (visuomotor coordination area); ROI #6 = right BA 1-3 (primary somatosensory cortices); ROI #7 = right BA 4 (primary motor cortex); ROI #8 = right BA 5 (somatosensory association area); ROI #9 = right BA 6 (premotor cortices); ROI #10 = right BA 7 (visuomotor coordination area).

#### Source Localization (e/sLORETA)

Exact Low Resolution Electromagnetic Brain Tomography (eLORETA) is a weighted L2 minimum-norm distributed source localization algorithm used for the estimation of three-dimensional current density in the brain from the measured scalp EEG spectral data (Pascual-Marqui et al., 1994; Pascual-Marqui, 2007). In the eLORETA (unit: μA/mm^2^) implementation, computations are made in a realistic head model using the MNI152 template, with the three-dimensional solution space restricted to cortical gray matter, as determined by the probabilistic Talairach atlas. The solution space consists of gray matter of the hemispheres and hippocampus (6,239 voxels at 5 mm grid in the MNI coordinate system). The specific frequency band cross-spectra (frequency-domain) obtained from the average-referenced scalp potential data, were the inputs for source localization. In summary, the cortical current density estimates from the 16 EEG electrode signals are assigned to an atlas space to obtain regions-of-interest (ROIs) based signals representing the source signals reflected in the recorded scalp EEG signal characteristics.

Overall, segments of clean EEG data (left/impaired hand movements and rest), separately for Pre- and Post-BCI intervention screening sessions, were used for the computation of cross-spectra (Mu [8–12 Hz] and Beta [18–26 Hz] bands) (Remsik A. B. et al., 2019). Averages of cross-spectra (1 average per each subject (*n* = 16), separately for movements and rest trials) were computed, and then eLORETA estimates of Mu [8–12 Hz] band power at 6239 cortical locations/voxels were obtained, using subject-wise normalization of the estimates (which means dividing the eLORETA current density estimate values by the grand-average over all voxels and frequency bands evaluated). Finally, the derived (movement – rest) eLORETA normalized estimates were used in paired-sample statistics (using statistical non-parametric mapping approach (SnPM) as implemented in the s/eLORETA software; voxel-based comparison).

Source-localized ERD in cortical space was chosen as a means of representing scalp-recorded brain signal changes during attempted hand movements. Activity recorded on the scalp is a representation of multiple source generators within cortical space. Extrapolating from the scalp to cortical space provides the justification for examining Mu ERD, an accepted brain signal associated with planning and executing movement (Pineda, 2005), and offers a means for visualizing surface EEG recordings in three dimensions. In fact, estimated source signals are a better representation of the underlying cortical generators that produce the activity recorded by scalp electrodes (Yuan et al., 2008).

#### Isolated Effective Coherence

Isolated effective coherence (iCoh) is a metric for frequency-specific directional flow of information transmission and offers a means of assessing direct paths of intracortical causal information flow of oscillatory activity. iCoh is based on formulating a multivariate autoregressive model from time series measurements and calculating the corresponding partial coherences after setting all irrelevant connections to zero, according to Pascual-Marqui et al. (2014). From the spectral density matrix (bandwidth of 4–30 Hz, with spectral resolution of 0.25 Hz, including Mu [8–12 Hz] and Beta [18–26 Hz] ranges) obtained from estimated signals in the selected ROIs, the partial coherences between any pair of nodes/ROIs can be calculated. The t-statistics was performed for iCoh values between post and pre-intervention screening session trials of the impaired (left) hand movement with threshold set at *p* = 0.05 (*t* = 2.13, uncorrected for multiple comparisons).

#### Hand Grip Function

Hand grip strength was assessed with a dynamometer (Boissy et al., 1999). Participants were asked to squeeze the spring-loaded dynamometer lever as hard as possible (i.e., maximal single hand grasp) with their entire hand, and then release. Three trials were performed with the affected hand and the average of the three trials was recorded as a handgrip score in pounds (lb).

### Statistical Analysis

Statistical differences between post- and pre-intervention cortical estimates were tested using paired-sample t-statistics as part of the statistical non-parametric mapping approach (SnPM) implemented in the s/eLORETA software (voxel-based). Paired-sample t-statistics were also used to test differences in the ROI estimates (power and connectivity). Finally, the Pearson correlation coefficient was used to quantify the correlation between change in hand grip strength and change in Mu rhythm desynchronization (Post vs. Pre BCI intervention). The *p* values reported are uncorrected for multiple comparisons.

## Results

### Localization of Mu ERD Changes Following Brain-Computer Interface Intervention

Exact Low Resolution Electromagnetic Brain Tomography (eLORETA) was used to estimate the three-dimensional current density in the brain from the measured scalp EEG spectral data. In the eLORETA implementation, computations are made in a realistic head model using the MNI152 template, with the three-dimensional solution space restricted to cortical gray matter, as determined by the probabilistic Talairach atlas.

**FIGURE 3:**
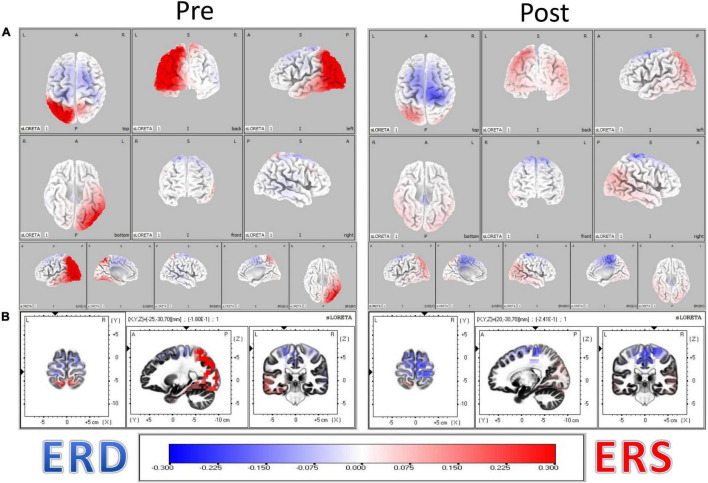
**(A)** Localization of Mu rhythm desynchronization of cerebral cortical sensorimotor areas during movement of the affected hand compared to rest. Computations are made in a realistic head model using the MNI152 template, with the three-dimensional solution space restricted to cortical gray matter, as determined by the probabilistic Talairach atlas. The specific frequency band cross-spectra (frequency-domain) obtained from the average-reference potential data were the inputs for source localization. The resulting distribution of ERD and ERS heatmaps illustrate the distribution of ERD and ERS changes from Pre to Post BCI intervention in the *n* = 16 stroke survivors. **(B)** Obtained eLORETA of Mu band power estimates at 6239 cortical locations (i.e., voxels) normalized across subjects.

**FIGURE 4:**
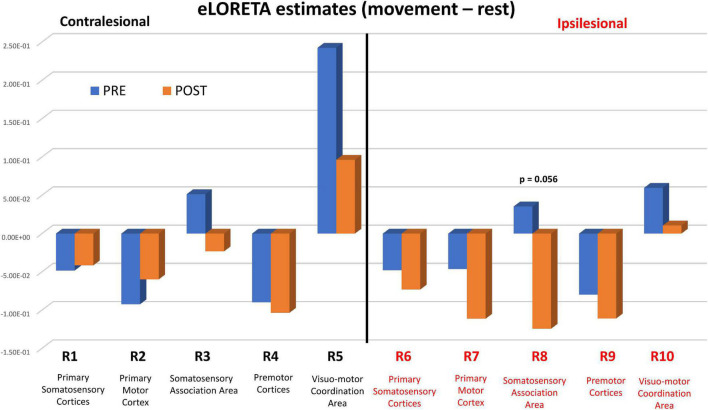
eLORETA estimates PRE (blue bars) and POST (orange bars) BCI intervention. eLORETA estimates between the two conditions [Movement – Rest] were based on measured EEG spectral power data in the Mu band [8–12 Hz] for each of the 10 regions of interest (ROI #1-10). P values refer to statistical group comparisons of Post vs. Pre estimates using a ROI-based one-tailed *t*-test. The *p* values reported are uncorrected for multiple comparisons.

The resulting distributions of ERD and ERS heatmaps of Figure 3 illustrate the distribution of ERD and ERS changes from Pre to Post BCI intervention in the *n* = 16 stroke survivors represented in Figure 4. Considering the distribution of ERS (red) and ERD (blue) voxels in Figure 3, it is apparent that as a result of BCI intervention, participants realized an increase of Mu ERD in ipsilesional hemisphere voxels of sensorimotor cortices during task performance (i.e., hand grasping) with the affected (left) hand compared to rest.

Figure 4 shows pre-BCI intervention (blue) and post-BCI intervention (orange) computed eLORETA cortical spectral power estimates for the Mu [8–12 Hz] band (i.e., 1 average per each of the 16 subjects included in these sub analyses, separately for movement (left) and rest trials) for each of the ten regions of interest and assumed underlying Brodmann’s Areas (Figure 2). ERD increases (Post vs. Pre BCI intervention) were observed in regions of interest ROI #6 through ROI #9. The largest increases in Mu ERD were observed for regions of interest ROI #7 and ROI #8, which correspond to ipsilesional primary motor cortex (BA 4) (paired-samples t-test: *p* = 0.11) and ipsilesional somatosensory association area (BA 5) (paired-samples t-test: *p* = 0.056), respectively (Figure 4).

### Correlation Between Mu Desynchronization and Hand Grip Strength

**FIGURE 5:**
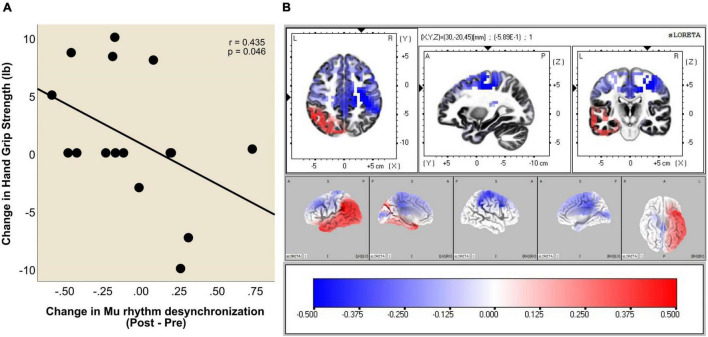
**(A)** Increase in Mu rhythm desynchronization in ROI #7 (i.e., ipsilesional primary motor cortex), Post vs. Pre BCI intervention, is significantly correlated with increase in hand grip strength (Pearson correlation coefficient *r* = 0.435, *p* = 0.046). Two data points overlap at (−0.20, 0.00). **(B)** Voxel-wise correlation of Hand Grip change with the Mu [8–12 Hz] rhythm desynchronization. The heat maps show colored *r* values (threshold set at *r* = 0.5). Scale: −0.5 (blue) to 0 (white) to + 0.5 (red). Note that stronger ipsilesional (R) Mu rhythm desynchronization (negative values) (Post-Pre) correlates with larger increase in hand grip strength.

Figure 5A plots change in hand grip strength as a function of change in Mu rhythm desynchronization (Post vs. Pre BCI intervention) in the primary motor cortex of the ipsilesional hemisphere (ROI #7). Each data point represents an individual subject. Please note that negative numbers in the change in Mu rhythm desynchronization (i.e., Post-Pre), represent higher desynchronization in the Post phase. That is, larger negative numbers subtracted from smaller negative numbers will yield negative numbers. Improved hand grip function is positively correlated with increased Mu rhythm desynchronization (Post-Pre) in the ipsilesional primary motor cortex (Pearson’s correlation coefficient *r* = 0.435, *p* = 0.046).

A voxel-wise correlation approach (refer to Methods section) was used to represent this relationship in brain atlas space (Figure 5B). The heatmaps show colored *r* values (e.g., Pearson’s correlation coefficient values) and indicate that the correlation between improvement in hand grip strength and increase in Mu rhythm *r* values is strongest in voxels representing ipsilesional cerebral cortical sensorimotor areas.

In summary, the above results suggest that for the stroke-lesioned hemisphere, BCI intervention facilitates increased Mu desynchronization associated with movement rehabilitation of the impaired upper extremity.

### Frequency-Specific Directional Flow of Information Transmission in BA 1-7

Direct paths of Pre to post BCI intervention frequency-specific flow of information transmission between cerebral cortical sensorimotor areas were analyzed by computing isolated effective coherence (iCoh) developed by Pascual-Marqui et al. (2014).

**FIGURE 6:**
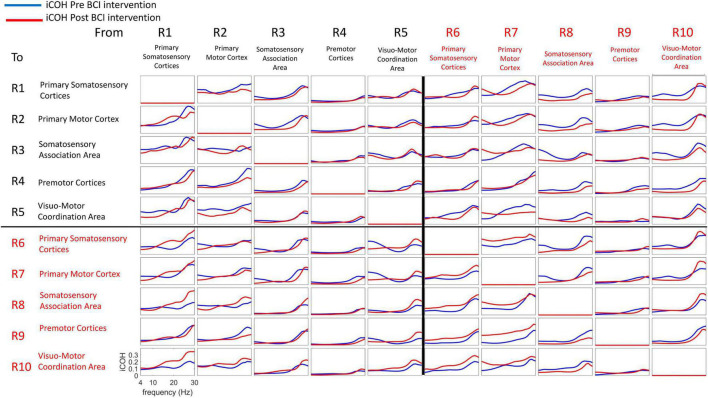
Isolated effective coherence (iCoh) – Frequency Specific Directional Flow of Information Transmission (Left Hand, Impaired Movement). Isolated effective coherence (iCoh), Pre (blue) and Post (red) BCI intervention. Vertical axis: 0 to 1. Horizontal axis: spectral frequency, 4–30 Hz. Columns are the senders, rows are receivers (i.e., FROM the top row of ROIs TO the horizontal columns of ROIs).

Figure 6 plots iCoh values as a function of spectral frequency, Pre (blue) and Post (red) BCI intervention, to and from the 10 regions of interest (ROI #1-10) for the affected (left) hand. Mean values (Pre, Post) were compared via paired-sample t-statistics, thresholded at *t* = 2.13 [*df* = 15], *p* = 0.05 uncorrected).

**FIGURE 7:**
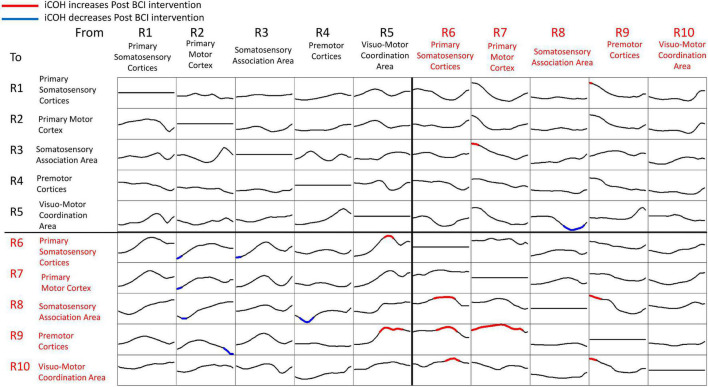
Plots of t-statistics for isolated effective coherence (iCoh) values Post vs. Pre BCI intervention of left (i.e., impaired hand) movement trials. Vertical axis: 0 to 1. Horizontal axis: spectral frequency: 4–30 Hz. Columns are the senders, rows are receivers (i.e., FROM the top row of ROIs TO the horizontal columns of ROIs). Red portions of the iCoh between two ROIs denote coherence increases at relative frequencies from Pre to Post, whereas blue portions denote significant decreases in iCoh from Pre to Post between two ROIs. Mean values (Pre, Post) compared via paired-sample *t*-statistics, with threshold set at *t* = 2.13 [df = 15], *p* = 0.05, uncorrected for multiple comparisons.

Figure 7 summarizes t-statistics for the iCoh data shown in Figure 6. Statistically significant increases and decreases in iCoh at relative frequencies from Pre to Post BCI intervention between pairs of ROIs are plotted in red and blue, respectively. Significant increases in iCoh from Pre to post BCI intervention were seen going from ROI #7 to ROI #9, suggesting more causal informational flow is going from ROI #7 (ipsilesional primary motor cortex) to ROI #9 (ipsilesional premotor cortices) Post compared to Pre BCI intervention. Significant decreases in iCoh were seen going from ROI #8 to ROI #5, suggesting less causal informational flow is going from ROI #8 (ipsilesional somatosensory association area) to ROI #5 (contralesional visuomotor coordination area) Post compared to Pre BCI intervention.

**FIGURE 8:**
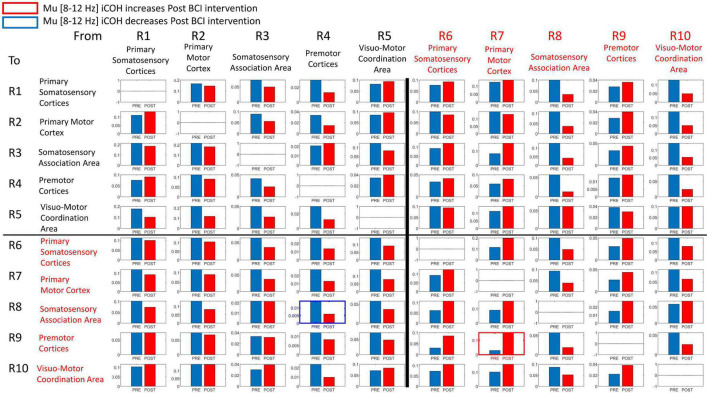
Plots of iCoh between two given ROIs in the Mu frequency band [8–12 Hz], Pre and Post BCI intervention. Vertical axis: Coherence value –1 to 1. Horizontal axis: Pre (blue) and Post (red). Columns are senders, rows are receivers (i.e., FROM the top row of ROIs TO the horizontal columns of ROIs). Blue surrounding boxes indicate significant decrease in iCoh while red surrounding boxes denote significant increases in iCoh (Post vs. Pre).

**FIGURE 9:**
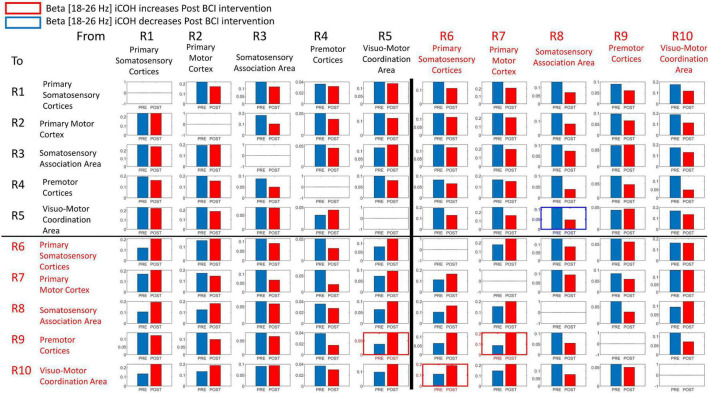
Plots of iCoh between two given ROIs in the Beta frequency band [18–26 Hz], Pre and Post BCI intervention, Vertical axis: Coherence value –1 to 1. Horizontal axis: Pre (blue) and Post (red). Columns are senders, rows are receivers (i.e., FROM the top row of ROIs TO the horizontal columns of ROIs). Blue surrounding boxes indicate significant decrease in iCoh while red surrounding boxes denote significant increases in iCoh (Post vs. Pre).

Signals in both Mu [8–12 Hz] and Beta [18–26 Hz] frequency ranges served as input commands in the present BCI design (Remsik A. B. et al., 2019). Figures 8, 9 summarize for Mu [8–12 Hz] and Beta [18–26 Hz] signal ranges, respectively, changes in functional connectivity of sensorimotor cerebral cortices during attempted movement of the impaired upper extremity as a result of BCI intervention. Each of the panels of Figures 8, 9 compares [Pre vs Post] iCoh values in Mu (Figure 8) and Beta (Figure 9) frequency ranges and represents a direct path of intracortical causal information flow of oscillatory activity between two ROIs. Statistically significant differences between Pre (blue bars) and Post (red bars) iCoh values between pairs of ROIs are represented by surrounding boxes with blue denoting significant decreases in iCoh, and red boxes signifying significant increases in iCoh.

In the Mu [8–12 Hz] frequency band, iCoh values significantly decreased Pre vs Post BCI intervention from ROI #4 (contralesional premotor area) to ROI #8 (ipsilesional somatosensory association area) (Figure 8). Mu band iCoh values significantly increased Pre vs Post BCI intervention from ROI #7 (ipsilesional primary motor) to ROI #9 (ipsilesional premotor cortices).

In the Beta [18–26 Hz] frequency band, iCoh significantly decreased Pre vs. Post BCI intervention from ROI #8 (ipsilesional somatosensory association area) to ROI #5 (contralesional visuo-motor coordination area) (Figure 9). Beta band iCoh values significantly increased Pre vs. Post BCI intervention from ROI #5 (contralesional visuo-motor coordination area) to ROI #9 (ipsilesional premotor cortices), from ROI #6 (ipsilesional primary somatosensory areas) to ROI #10 (ipsilesional visuo-motor coordination area), and from ROI #7 (ipsilesional primary motor cortex) to ROI #9 (ipsilesional premotor cortices) (Figure 9).

## Discussion

We have investigated potential neural substrates underlying the effect of BCI intervention on motor recovery in stroke survivors. Here we report, for grasping movements of the affected hand compared to rest, significantly greater desynchronization of sensorimotor rhythms in the Mu frequency band [8–12 Hz] recorded via scalp electrodes in the ipsilesional hemisphere, indicating greater activation of the ipsilesional sensorimotor system following BCI intervention. Moreover, we demonstrate that the increased Mu desynchronization in the ipsilesional primary motor cortex, Post vs. Pre BCI intervention, correlates significantly with improvements in hand function as assessed by grip force measurements. Furthermore, analyses of frequency-specific directional flow of information transmission between cerebral cortical sensorimotor areas, deduced from measurements of isolated effective coherence (iCoh), revealed both intra- and interhemispheric changes as a result of BCI intervention, in particular, increased functional connectivity from ipsilesional primary motor to ipsilesional premotor cortices, and from contralesional visuo-motor coordination area to ipsilesional premotor areas, respectively.

A growing body of evidence supports the effectiveness of EEG-based BCIs on improvement of upper extremity motor function following stroke (for meta-analyses/reviews see Cervera et al., 2018; Bai et al., 2020; Simon et al., 2021). BCI paradigms utilizing FES and/or attempted voluntary movements of the hemiparetic hand are particularly promising interventions for the rehabilitation of stroke survivors (Ramos-Murguialday et al., 2013; Kim et al., 2016; Biasiucci et al., 2018; Nishimoto et al., 2018; Remsik et al., 2018; Tabernig et al., 2018) because they may induce and/or facilitate neuroplastic changes, at both structural and functional levels, that link movement intention with muscle contraction (Daly and Wolpaw, 2008; Ramos-Murguialday et al., 2013; Biasiucci et al., 2018; Bai et al., 2020).

### Source Localization of Mu ERD in Sensorimotor Cortices

In a less tightly controlled analysis of available NCT02098265 participants at the time, our laboratory demonstrated (Remsik A. et al., 2019) that ipsilesional Mu [8–12 Hz] ERD increases, recorded over cerebral cortical sensorimotor areas by scalp electrodes C3/C4 (Figure 1), were correlated with motor recovery of the affected upper extremity in stroke survivors. Here, we confirm and expand upon our previous findings in three ways. First, we present a more detailed source mapping to cortical space of Mu rhythm desynchronization and show that motor recovery is predominantly associated with Mu ERD changes ipsilesionally, near the primary motor cortex (Brodmann Area (BA) 4) and somatosensory association area (BA 5) (Figure 3). Second, we show that greater Mu desynchronization in ipsilesional primary motor cortex (BA 4) is significantly correlated with improvement in hand grip strength, an objective, quantitative measure of UE function and capacity, following BCI-FES intervention (Figures 4, 5). Third, we show evidence of functional connectivity changes between ipsilesional sensorimotor cortices following BCI-FES intervention, which is consistent with the view that BCI-FES use supports and/or engages fundamental neural mechanics that link volitionally controlled EEG signal changes with changes in objective behavioral measures of motor function and capacity. Such mechanisms may be specific to motor recovery (Biasiucci et al., 2018; Bai et al., 2020) and/or may be signatures of motor learning in general (Yuan et al., 2008; Edelman et al., 2019).

Our results are in line with findings of other studies that have employed similar BCI paradigms in cohorts of stroke survivors with similar demographics (Daly et al., 2009; Ramos-Murguialday et al., 2013; Ang et al., 2014; Ono et al., 2014; Popovic, 2014; Hu et al., 2015; Kim et al., 2016; Biasiucci et al., 2018; Nishimoto et al., 2018; Tabernig et al., 2018; Bai et al., 2020; Simon et al., 2021). For example, Biasiucci et al. (2018) report that only their BCI-FES group exhibited significant functional improvement following BCI intervention. Our result that increased ipsilesional Mu desynchronization is significantly correlated with increased grip strength is in good agreement with the finding of Biasiucci et al. (2018) that only their BCI-FES group exhibited a significant and lasting increase of the strength of the muscle targeted by the FES (in their study: extensor digitorum communis, which elicits full extension of the wrist and fingers). Furthermore, our result that SMR desynchronization is largely ipsilesional, indicating greater activation of the ipsilesional motor system following BCI-FES intervention, is in keeping with previous reports in stroke survivors (Mihara et al., 2013; Li et al., 2014; Biasiucci et al., 2018; Bai et al., 2020). BCI-FES devices may interface with native underlying mechanisms of neuromotor plasticity and control that have been demonstrated to engage and facilitate brain changes and behavior changes indicative of clinical motor recovery.

### Functional Connectivity and Motor Recovery

Importantly, although voxel-wise distribution of eLORETA output revealed a sensorimotor-centered focus of Mu ERD change associated with motor recovery, ipsilesional-focused Mu ERD change may not be the sole driver of motor recovery in stroke survivors using a BCI. Mu signal changes in cortical sensorimotor areas may be part of a broader integration of disparate ROIs in a neural network that includes both intra- and interhemispheric components (Figures 8, 9), although recovery of the primary motor region has been demonstrated to be essential to motor recovery (Grefkes and Fink, 2014). Therefore, we sought to understand whether the underlying neural network is specific to localized neural populations represented by the Brodmann’s area segmentations or, whether there is a larger geography of populations – functionally connected neural networks – that may be influenced by participation in the BCI intervention. We used discrete data in time series to represent directionality or sequencing of activity associated with these changes to test the hypothesis that BCI intervention results in changes in frequency-specific directional flow of information transmission (functional connectivity) in ipsilesional and/or contralesional sensorimotor areas (Ward et al., 2003; Rehme et al., 2011; Dubovik et al., 2012; Várkuti et al., 2013; Nicolo et al., 2015; Pichiorri et al., 2015; Pundik et al., 2015; Wu et al., 2015; Biasiucci et al., 2018).

The changes in functional connectivity reported here, particularly between ipsilesional primary motor cortex and ipsilesional premotor cortices, agree with previous studies that have employed comparable BCI designs (Wu et al., 2015; Biasiucci et al., 2018). Increased interactions between ipsilesional motor brain areas are associated with better recovery and motor performance (Dubovik et al., 2012; Nicolo et al., 2015; Wu et al., 2015). The change in effective connectivity between ipsilesional motor cortex and premotor cortices following BCI intervention was observed for both Mu and Beta frequency ranges in both the present results (Figures 8, 9) and those of Biasiucci et al. (2018), which is consistent with the view that the functional improvement following BCI intervention is due to facilitation and induction of neuroplastic changes associated with motor recovery as well as motor learning more broadly.

### Brain-Computer Interface-Functional Electrical Stimulation and Motor Recovery

BCI-mediated FES leads to the most significant recovery of motor function following stroke (Bai et al., 2020; Simon et al., 2021). The inclusion of FES is thought to contribute to the clinical effect of a BCI not only through the somatosensory contribution of facilitated muscle stimulation but also through pairing of volitionally modified CNS efferent signals with stimulation (i.e., activation) of the impaired distal muscle (Bergquist et al., 2011). Biasiucci et al. (2018) have demonstrated through their BCI-FES versus sham FES experimental design that it is not the FES alone but rather the contingency between rich sensory feedback and suitable activation of cortical motor areas that may drive activity-dependent, Hebbian plasticity that may underlie motor recovery in BCI-FES interventions. Whereas our study design does not allow us to draw the same conclusions as Biasiucci et al. (2018) with respect to the clinical significance of the FES adjuvant, both our results and BCI-FES device are similar to those of Biasiucci et al. (2018). Therefore, it is likely that the clinically relevant functional gains demonstrated here are due to the same strict contingency of BCI-driven FES detailed by Biasiucci et al. (2018).

Future research is needed to better identify and track the genesis and progression of neuroplastic changes, and to determine the relative importance of the various intra- and interhemispheric network connectivity changes presented here. In addition, further research is necessary to discover the mechanistic origins of any such neuroplasticity, and how such mechanism(s) may improve rehabilitation strategies that enable caregivers to provide maximal benefit to patients (Bai et al., 2020; Simon et al., 2021).

### Limitations

Localizing cortical neuronal signatures with EEG scalp electrodes is a process that neuroscience researchers and others have used for some time (Pascual-Marqui et al., 1994; Pascual-Marqui, 2007). It is generally believed that higher density electrode arrays in the EEG cap provide more reliable and accurate localization estimations of the underlying neurophysiological processes. The use of eLORETA in a 16-channel cap arrangement, in non-research settings, likely pushes the boundaries of this method’s practical limits as an estimation tool for source localization of motor-EEG brain signals. Co-registration of spectral EEG data to MNI atlas space is a first step toward addressing this issue. In this work, scalp electrodes, while limited in number (16 channels), specifically covered sensorimotor cortices with greater density than the conventional 10–20 electrode placement system. Arguments can also be made for the use of “individualized” head modeling over “standard” head models. The use and reference to Brodmann’s areas in this work are intended solely as representative labels of the brain segmentations proposed by Brodmann, and we assume that these areas are generally emblematic of the functional cortical brain areas recorded by the scalp EEG electrodes of this cap arrangement (Figure 1).

In future research, repeating or initiating a similar BCI study with high density or 19 channel standard electrode arrays might allow a more strict evaluation of the “boundaries” in channel count and “electrode density as spatial coverage over areas of interest” by comparing the source localization results from these findings to the aforementioned high- and low-density electrode setups (i.e., with “full” head coverage). With any channel count placed “strategically” with higher density than the basic *n* = 19 (i.e.,10-20 system) over local functional network areas of interest on the scalp, reliable recordings of neuro-electrophysiological functioning might be feasible.

## Conclusion

The results of the present study are consistent with the view that EEG-based BCI-FES intervention enhances information flow between cerebral cortical sensorimotor areas involved in motor planning, motor execution, and motor learning, and as such aides in establishing BCI-FES intervention as an effective therapy for motor rehabilitation of stroke survivors.

BCI-FES intervention may help facilitate adaptive brain changes, such as increased ERD during movement of the impaired upper extremity in the stroke-lesioned brain, but it is the brain’s ability to adapt its functional connections (i.e., plasticity of the sensorimotor system) that may underlie the potential of BCI-FES intervention as a rehabilitation strategy.

In conclusion, this study not only helps to establish the efficacy of BCI-FES intervention as a therapy for stroke survivors but is also important for increasing our understanding how sensorimotor processing contributes to the transformations of plans for voluntary limb movements into muscle commands necessary for their execution in healthy individuals. Thus, the results have the potential to guide development of innovative strategies for motor rehabilitation and are also important for increasing understanding of motor control in general.

## Data Availability Statement

The original contributions presented in the study are included in the article/supplementary material, further inquiries can be directed to the corresponding author/s.

## Ethics Statement

The studies involving human participants were reviewed and approved by University of Wisconsin Health Sciences Institutional Review Board. The patients/participants provided their written informed consent to participate in this study.

## Author Contributions

AR involved in subject recruitment, staff training, data collection, data processing, data analysis, interpreting data, and writing of the manuscript. KG involved in analysis, interpreting writing, and editing of the manuscript. LW involved in staff training, data collection, data processing, data analysis, interpreting data, and writing of the manuscript. PK, SG, and EB contributed to writing of the manuscript, manuscript editing, and intellectual content. CR and SR involved in data collection and editing the manuscript. BY involved in subject recruitment, staff training, data collection, data processing, data analysis, interpreting data, intellectual content, and manuscript editing. VN contributed to subject recruitment, staff training, data collection, data processing, data analysis, interpreting data, manuscript editing, and intellectual content. KC, JW, and VP are co-PIs and were involved in study conception, design, staff training, subject recruitment, manuscript editing, intellectual content, and supervised all aspects of the study. All authors contributed to the article and approved the submitted version.

## Conflict of Interest

The authors declare that the research was conducted in the absence of any commercial or financial relationships that could be construed as a potential conflict of interest.

## Publisher’s Note

All claims expressed in this article are solely those of the authors and do not necessarily represent those of their affiliated organizations, or those of the publisher, the editors and the reviewers. Any product that may be evaluated in this article, or claim that may be made by its manufacturer, is not guaranteed or endorsed by the publisher.
